# Can Parametric Microinsurance Improve the Financial Resilience of Low-Income Households in the United States? A Proof-of-Concept Examination

**DOI:** 10.1007/s41885-021-00088-1

**Published:** 2021-07-30

**Authors:** Carolyn Kousky, Helen Wiley, Len Shabman

**Affiliations:** 1 Wharton School, University of Pennsylvania, Philadelphia, PA USA; 2 Resources for the Future, Washington, DC USA

**Keywords:** Insurance, Microsinsurance, Disasters, Parametric insurance

## Abstract

Natural disaster risk is escalating around the globe and in the United States. A large body of research has found that lower-income households disproportionally suffer from disasters and are less likely to recover. Poorer households often lack the financial resources for rebuilding, endangering other aspects of wellbeing. Parametric microinsurance has been used in many developing countries to improve the financial resilience of low-income households. This paper presents a review of the evidence for implementing parametric microinsurance in the U.S., with spillover lessons for other highly developed countries. We discuss the benefits and the challenges of microinsurance in a US context and explore 4 possible distribution models that could help overcome difficulties, with policies being provided: (1) by an aggregator, (2) through a mobile-based technology, (3) by linking to other products or retailers, or (4) through a public sector insurer.

## Introduction

Natural disaster risk is escalating around the globe and in the United States (Gall et al. 2011; Hoeppe 2016; USGCRP 2018; Coronese et al. 2019). With longer and more intense wildfire seasons, record numbers of acres and structures are being burned (Abatzoglou and Williams 2016; Williams et al. 2019). Coastal communities are facing rising flood risks as storm patterns shift and sea levels rise (e.g., Garner et al. 2017; Sweet et al. 2020). Localized extreme weather events are becoming more frequent with costly consequences (Stott 2016). Earthquakes continue to pose significant risks for portions of the U.S., with more people living or working in areas of high or moderate seismic hazard than ever before (Petersen et al. 2020). In addition, compound and cascading hazards are increasing (Cutter 2018; AghaKouchak et al. 2020). The consequences of these rising disasters, however, are not borne evenly.

A growing body of research—reviewed below—finds that the poor are disproportionately harmed from disasters, both worldwide and in the United States (Fothergill and Peek 2004; Hallegatte et al. 2020). A natural disaster is a negative economic shock—an event of limited duration where income declines and/or necessary expenditures increase. Lower-income households typically do not have sufficient liquid savings to fund the necessary repairs and recovery. These households are also often locked out of access to credit (Collier and Ellis 2020). Governmental aid programs, contrary to some misconceptions, are typically inadequate and often extremely delayed, leaving households suffering for weeks, months, or even years before funds arrive. They also offer little assistance that is means-tested or specifically targeted to help lower-income households.^1^ Lower-income households are usually uninsured, as indemnity-based disaster policies available in the U.S. are unaffordable for them.

These constraints on post-disaster financing have spillover impacts for all aspects of life. Having timely access to funds needed to rebuild and repair damages is linked to emotional well-being, physical health, mental health, educational attainment, and the stability of families (Farrell and Greig 2018; McKnigh 2019). Without the resources to recover, households turn to coping mechanisms that can have long-term negative impacts and limit their ability to build wealth (Jacobsen et al. 2009). This is a challenge for many families in the U.S. In 2020, the poverty rate, as defined and measured by the U.S. Census for the contiguous U.S., was roughly 12% and was increasing through the year as a result of the economic impacts of COVID-19 (Parolin et al. 2020). An even larger percentage of households are lower-income but above the federal poverty line.

Insurance can be made more affordable for lower-income households through three primary channels: (1) coverage levels could be reduced or the policy designed for less frequent events, (2) administrative and transaction costs could be reduced and the savings passed on to consumers, or (3) a direct public subsidy could be provided. Parametric microinsurance has the potential to increase the financial resilience of lower-income households by potentially harnessing all three channels. Microinsurance refers to insurance policies that have low premiums and lower coverage limits and are designed to serve lower-income populations. Lower-income consumers typically need smaller coverage levels, harnessing the first channel. Further reductions in coverage or limiting the range of events for which the policy provides payouts, however, would also dramatically reduce the benefits of the policy and thus attempts to make insurance affordable solely through the first channel can undermine the benefits of the insurance. Parametric insurance refers to insurance policies that rapidly pay a set amount based on observable measures of the disaster, such as wind speed in a certain location (Sengupta and Kousky 2020). When the designated parameter is reached, a payout is triggered. Parametric insurance typically has lower transaction costs, since there is no need for costly and time-consuming loss adjusting; underwriting costs are lower since often all that is needed is a location; and claims management can be lower, since there tend not to be difficult legal disputes. As such, a parametric structure can harness the second channel, lowering premiums by passing on savings in reduced transaction costs. Finally, as discussed in the paper, many existing microinsurance programs in the world also benefit from some type of public or philanthropic support—the third channel.

To date, almost all applications of parametric microinsurance policies come from developing countries, where such products have increasingly been piloted and implemented. Parametric microinsurance coverages have included life and health, as well as crop and livestock insurance to help smallholders with weather-related losses (e.g., Barnett et al. 2008).^2^ Microinsurance has not often been used to improve the post-disaster financial resilience of lower-income populations in developed nations. This paper investigates the potential for parametric microinsurance to support post-disaster recovery of lower-income households in the United States, where limited income and assets, limited ability to borrow, and limited programs of disaster aid targeted to lower income households have to-date impeded recovery for this population.

The paper provides a review of the evidence on whether and how the concept of parametric microinsurance can be extended to the U.S. Our assessment of the potential for bringing parametric microinsurance to a developed country context begins with an extensive review of the literature on both the post-disaster recovery needs of lower-income households and on developing nations’ experience in order to identify lessons learned. This includes an investigation of the role of the public sector and philanthropy in making microinsurance viable. We then conducted semi-structured interviews with regulators, insurers, and recovery experts to identify the challenges and opportunities for offering parametric microinsurance in the U.S. Until 2021, there had been no products marketed as microinsurance offered in the U.S. In the summer of 2020, though, Puerto Rico’s insurance commissioner introduced regulations to enable the launch of parametric microinsurance products to protect low-income families from natural disasters. At least one firm, Raincoat, began offering such a product in the spring of 2021.

The paper is structured as follows. The second section begins with a review of the literature on the disaster recovery challenges facing low-income households in the U.S.—the target population for parametric microinsurance. The third section provides an overview of parametric microinsurance and its potential benefits. The fourth section discusses the challenges of implementing parametric microinsurance both broadly and specifically in the U.S. The fifth section describes four distribution models for how parametric microinsurance could be adopted in the U.S., with policies being provided: (1) by an aggregator, (2) though a mobile-based technology, (3) by linking it to other products or retailers, or (5) by a public sector insurer. The sixth section concludes with reflections on next steps for exploring applications.

## The Recovery Needs of Low-Income Households in the United States

### Disproportionately Harmed

A mix of qualitative and quantitative studies across different locations in the United States all find that lower income groups and minorities, as is the case worldwide, suffer disproportionately in the aftermath of a disaster and recovery is slower than more privileged residents and may not be complete (Bolin and Bolton 1986; Fothergill et al. 1999; Tierney 2006; Brunsma et al. 2010; Fussell and Harris 2014). Aggregate loss statistics or examination of macroeconomic indicators can mask this reality (e.g., Kim 2012; Hallegatte et al. 2017; Sawada and Takasaki 2017). Follow-on economic impacts, such as declines in credit scores, are not only more severe in low-income communities and communities of color, but can persist for years (Ratcliffe et al. 2019). Without financial safety nets, disasters can become tipping points into deeper poverty, as families and individuals default on loans, accumulate debt, exhaust small savings for other purposes like education, or even lose ownership of their homes (e.g., Fothergill and Peek 2004; Pastor et al. 2006).

Disasters add new and myriad additional expenses for households. These expenses range from immediate ones like paying for evacuation costs and temporary housing to longer-term expenses for repairing homes and businesses, replacing damaged goods, or permanently relocating. Even when there is not significant property damage, families might have to cover a number of unexpected costs for extended periods. For example, when electricity was lost for weeks to months in parts of Puerto Rico following Hurricane Maria in 2017, households had to purchase generators and fuel to keep their homes habitable when the grid was down (Fausset et al. 2017). When households lack other sources to cover these disaster costs, they may have to defer other expenses, such as healthcare and debt servicing, in order to have sufficient funds (Farrell and Greig 2018).

Wealth inequalities are substantial and increasing in the United States; this has effects on other dimensions of social stratification, including educational attainment, physical health, and emotional well-being (Hansen 2014; Keister 2014; Shapiro 2017). Historical disparities and existing inequities lead to uneven impacts from disasters (Finch et al. 2010) and compound social and wealth inequities (Howell and Elliott 2019). Prior research also demonstrates that low-income residents recover less quickly when compared with more-privileged residents, who may even benefit financially post-disaster (Brunsma et al. 2010; Fussell and Harris 2014). This slower recovery is typical not only for low-income households, but also those with specific racial or ethnic backgrounds and areas with lower cost rental units (Kamel and Loukaitou-Sideris 2004; Tafti and Romlison 2019). Hard-hit disaster areas are often characterized by slowed income and employment growth and economic activity may shift to the edges of disaster zones, where damage is minor. When recovery for a low income population depends on access to alternative forms of employment in the face of job instability (Tanner et al. 2015), these short-term shifts can permanently alter the spatial distribution of employment and income, possibly exacerbating wealth inequities (Xiao and Nilawar 2013).

Low-income renters face their own post-disaster challenges. Renting in the United States is at a 50-year high (Cilluffo et al. 2017), with 36% of households renting as of 2019, according to the U.S. Census Bureau.^3^ While renters are not responsible for building repairs, their possessions may be damaged. Beyond costs sustained from damaged possessions, renters may have to pay higher rents post-disaster if rents are raised to fund repairs or due to high demand for a reduced number of units (Peacock et al. 2014). If a disaster makes a rental unit uninhabitable, residents can effectively become evicted by a storm. Eviction carries with it numerous negative impacts for families (Desmond 2016). One study finds that local damages are more likely to impact the finances of low-income renters who may lose local jobs, need to relocate, and/or pay higher rents due to reduced housing stock, but have limited savings to draw on (Elliott and Howell 2017). Several additional factors can slow the recovery of rental housing, including that original construction materials for rental units, particularly multi-unit buildings, may be lower quality and poorly maintained and therefore subject to greater damage; costs of repair may be greater; and disaster assistance typically becomes available later for rental properties (Comerio 1998; Fussell 2015).

Table 1 summarizes the preceding discussion of the range of potential post-disaster financial consequences to low-income households identified in the literature review. Note, of course, that different households will have different disaster loss experiences depending on the particular event and their individual circumstances; they are unlikely to experience every cost in Table 1.

**Table 1:** Potential post-disaster financial impacts for lower-income households

Response Costs	Property Costs	Financial Impacts
Evacuation transportation	Repairing home	Decreased employment
Temporary lodging	Repairing/replacing contents	Consumption of savings
Additional meals	Relocation	Additional debt
Generator and fuel	Higher rent	Deferred expenses (e.g., healthcare)
Preparedness supplies	Debris clean-up	

### Insufficient Recovery Resources

Risk management and social protection are usually high in developed countries and they have well-developed insurance markets (Holzmann et al. 2003). Indeed, the United States and Canada accounted for over 57 percent of nonlife insurance premiums in the global market in 2019^4^ and the U.S. is the world’s largest insurance market. Despite having a mature insurance market, and despite every state in the country having been impacted by at least one billion-dollar disaster since 1980,^5^ a significant portion of the U.S. population is uninsured or underinsured for natural disasters, with low-income households, in particular, often not having coverage (e.g., Peacock et al. 2007; Insurance Information Institute 2014; FEMA 2018; Klein 2018). This is partially due to lower-income families often lacking in standard property or renters insurance, but also because those policies fully exclude some disasters, such as flood and earthquake, or put coverage limitations on others, such as hurricane wind damage, and purchasing additional coverage can be cost prohibitive.

This limited purchase of disaster insurance among lower-income families means they must rely on highly limited other sources of funds for disaster recovery: savings, credit, or governmental aid. Roughly 40% of households do not have $400 in liquid funds for an emergency (Board of Governors of the Federal Reserve System 2018). The first line of assistance for disaster victims in the U.S. is often a loan, yet credit typically fails for lower-income households as they may not have the resources to take on additional debt or may be locked out of access to loans altogether. Indeed, over half of applicants to the disaster loan program of the Small Business Administration—the federal loan program for disaster victims—are rejected as uncreditworthy because they do not meet debt-to-income or credit score requirements (Collier and Ellis 2020).

Without savings or a loan, households turn to aid. Non-governmental organizations (NGOs) may provide some needed support. For example, after Harvey, roughly 30% of respondents to a survey said they received some assistance from a national charity (Hamel et al. 2018). Since the availability and amount given by NGOs will vary and impossible to predict ex-ante, it is not an adequate or reliable source for recovery. Households may depend on the government, but contrary to many perceptions, federal disaster aid is often limited and delayed, making it, too, an insufficient recovery source. Federal assistance is only provided following large disasters that receive a Presidential disaster declaration. Even for these events, households may not receive funds. Between 2000 and 2020, grants to households from the Federal Emergency Management Agency (FEMA) were authorized in less than 30 percent of major disaster declarations.^6^ When provided, these household grants are capped and typically average only a few thousand dollars. According to FEMA, the program “is not a substitute for insurance and cannot compensate for all losses caused by a disaster; it is intended to meet basic needs” (FEMA 2016). Other potential sources of federal aid, such as programs financed by Congressional appropriations to the Department of Housing and Urban Development, are uncertain, and when funded, take many months, or more typically years, to get funds to households (Spader and Turnham 2014). They are designed for long-term recovery and hazard mitigation, not meeting the financial needs of households in the weeks and months following a disaster. An analysis of Hurricane Sandy, for example, found that there was the least assistance for immediate rebuilding, with negative long-term economic impacts for homeowners (Madajewicz and Coirolo 2016). There have also been concerns that these funds are distributed inequitably, favoring white and more affluent homeowners, and are not meeting the needs of renters and more disadvantaged residents (e.g., Capps 2020; FEMA 2020; Billings et al. 2021).

While in some situations, households may be able to turn to friends or family for assistance, in a disaster, entire neighborhoods may be hit simultaneously. As such, resilience typically requires that disaster risks be transferred out of the community (Jacobsen et al. 2009), but, as noted, this is rarely the case for low-income communities in the U.S. Insufficient and uncertain aid, coupled with unaffordable insurance premiums, has created an enduring disaster recovery gap for low-income households. This may explain why research on Hurricane Katrina documented that the recovery process itself further exacerbated inequalities for low-income and non-white residents compared to wealthier residents (Elliott et al. 2009; Adams 2013). A survey of survivors of Hurricane Harvey found that three months and twelve months post-hurricane, around 40% said they were not getting the help they needed for recovery; lower-income and Black respondents were more likely to say they are not receiving sufficient assistance (Hamel et al. 2018). The same study found that low-income households were less likely to say that insurance and aid would cover some of their losses and were more likely to have had to take on extra work or had fallen behind in payments due to insufficient recovery resources.

**Fig. 1:**
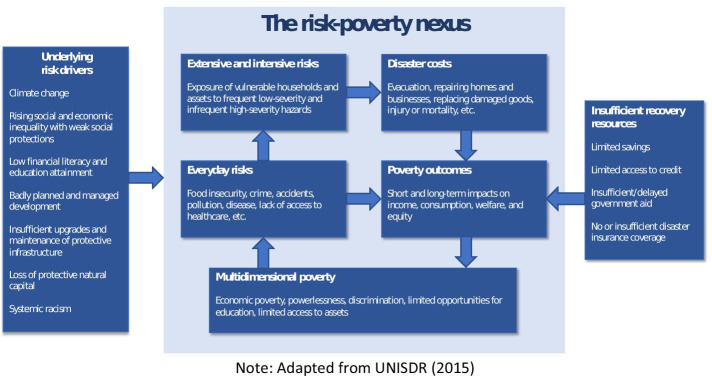
The Disaster-Poverty Cycle

Figure 1, adapted from the United Nations Office for Disaster Risk Reduction (UNISDR 2015), depicts the full range of interconnections between disaster risk and poverty. While having applications globally, these relationships also exist in the United States. A range of underlying risk drivers influence the complex relationship between risks and poverty. Insufficient recovery resources exacerbate the burden of disasters on poor households.

## Overview of Parametric Microinsurance and its Potential Benefits

**Fig. 2:**
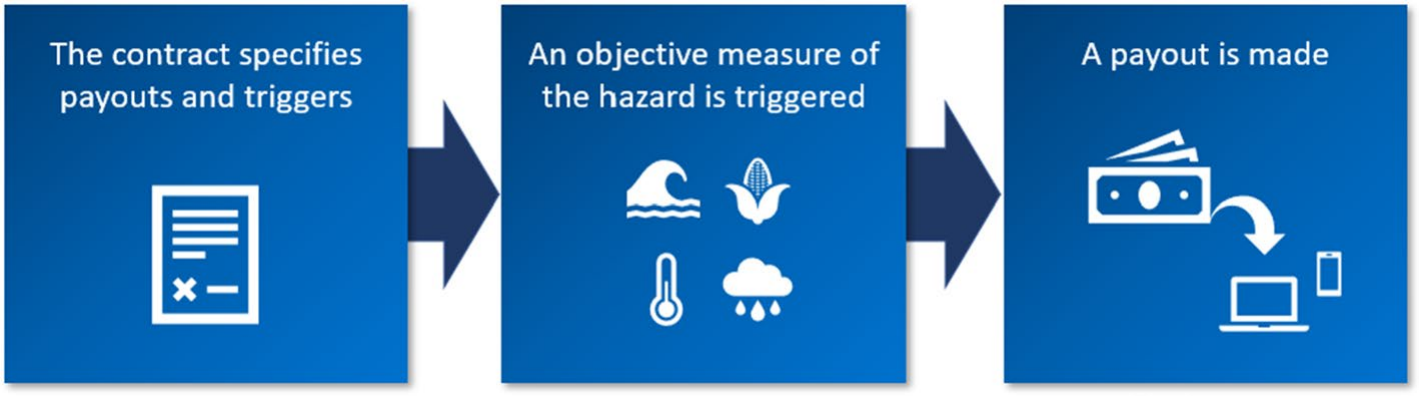
Parametric Insurance Overview

Microinsurance refers to low-coverage, low-premium insurance policies that are designed to protect low-income households from financial shocks. The International Association of Insurance Supervisors defines microinsurance as coverage “that is accessed by low-income population[s], provided by a variety of different entities, but run in accordance with generally accepted insurance practices,” (IAIS 2007, p.10). Microinsurance policies have been offered in the developing world for health insurance, life insurance, and agricultural insurance, among other lines. Given the targeted consumer, microinsurance must be affordable, simple, accessible, and the delivery process must be efficient (Churchill and McCord 2012). Due to these needs, microinsurance is almost always a parametric product (sometimes called index-based insurance). Parametric insurance pays the insured a set amount based on an objective measure of the severity of a specific event, instead of based on the amount of damage sustained (Sengupta and Kousky 2020). In the U.S., most consumers are more familiar with indemnity insurance, which would compensate the insured exactly for a loss (subject to deductibles and coverage limits). With a parametric policy for natural disasters, the policy contract specifies the amount paid for certain measures of the hazard, such as windspeed in a certain location or the water height at a set of stream gauges (see Fig. 2).

The indicator that determines the payout is referred to as the trigger (there could also be multiple triggers that must be met for payment). A well-designed trigger depends on objective data analysis that allows for rapid reporting and independent third-party verification. The trigger should also be highly correlated with the insured’s loss. The potential difference between the actual loss and the payout is referred to as basis risk, and is one downside of parametric insurance. Care must be taken in designing the policy to minimize the basis risk to the insured and clearly explain the payout structure. This is especially true for vulnerable populations that are dependent on the microinsurance funds for recovery. We discuss the challenge of basis risk further in the “Meeting Regulatory Requirements” section below. Basis risk, however, is a cost that must be accepted to secure the benefits of parametric insurance, which are often what enable microinsurance to be written in the first place. Indemnity-based insurance has administrative and transaction costs that add to the required premium revenue, making it too costly for microinsurance. Processing claims for a parametric product, on the other hand, does not require expensive and time-consuming adjusters and there can be fewer disputes. Underwriting costs may also be lower for parametric products, since it only requires modeling the hazard and not consideration of policyholder-level specifics that could influence losses.

Beyond lower transaction costs, parametric policies have two important benefits that also apply in the case of microinsurance. First, the ease in determining claims means that payouts are made much more rapidly with parametric products, often in a matter of days. Given the delays discussed above for other sources of recovery funds, this can be a critical role for these products. Second, parametric insurance also provides important flexibility to the insured. The funds can be used for any immediate need, many of which are hard to predict before the disaster occurs.

### The Role for Parametric Microinsurance in the U.S.

Parametric microinsurance is one approach to the development of inclusive insurance—often defined as any program or policy that makes insurance coverage available to those previously locked out of the insurance market. Parametric microinsurance is not going to be appropriate for all low-income households and all perils. It is, though, one more important tool in the toolbox for improving the financial resilience of vulnerable populations. In this section, we detail more specifically the role it could play in the U.S.

Our focus here is on coverage for disasters. Disasters are a covariate risk, meaning they are experienced by entire neighborhoods or communities at the same time. It is perhaps worth first noting that parametric microinsurance would not be appropriate for covering idiosyncratic risks, which impact just the individual. Parametric policies, by relying on third-party measures of disaster, are only appropriate for covering covariate risk since local or regional disasters would be more highly correlated with a third-party trigger. As such, households that face large idiosyncratic risks, in addition to any covariate risk, may not be well-suited for this type of policy. They may need a more comprehensive indemnity policy that would cover a wide range of risks. Unfortunately, in the United States, disaster perils are typically excluded from more comprehensive property policies and indemnity disaster insurance may exceed the ability to pay of lower-income households. As such, these households may not be able to afford insurance for both idiosyncratic and disaster shocks.

For example, FEMA has estimated that there are approximately 3.3 million homeowners and renters in the 100-year floodplain that do not currently have flood insurance (floods are excluded from homeowners policies), and that these households tend to have significantly lower income than those who do have flood insurance, suggesting affordability constraints play a role in who is covered for flood (FEMA 2018). More specifically, FEMA finds that 51% of the 3.3 million households are defined as extremely low, low, or very low income, according to definitions from the Department of Housing and Urban Development.^7^ In addition, there are many households at risk of flooding outside the FEMA-mapped 100-year floodplain, as well, that fall into these categories. A back-of-the-envelope calculation suggests, then, that there are at least 1.68 million households at risk of flooding that may struggle to pay for a standard indemnity flood policy and who could potentially benefit from a parametric microinsurance flood product.

Thus, while not comprehensive, microinsurance can be thought of as a product that lower-income households can afford, providing them some financial protection that is critical to recovery when the alternative is no insurance coverage at all. For instance, the Puerto Rican enabling regulation for microinsurance (discussed further below), notes that “it is intended to provide a financial protection tool for persons who otherwise could not purchase traditional insurance.” In addition, parametric microinsurance for disasters could provide substantial value in a number of other situations: for perils that will not fully destroy a home, for renters who are less concerned about property damages but will struggle with other post-disaster expenses, for mobile-home owners whose total needed payout would be lower,^8^ and to cover non-property disaster costs, including evacuation costs. In addition, as discussed above, there can be serious timing delays to the receipt of both governmental disaster assistance and indemnity-based insurance payouts (Paganini 2019): a parametric microinsurance policy could fill this timing gap in receiving needs funds.

There are, therefore, a range of important cases where parametric microinsurance has a key role to play in recovery. This does not mean, however, that it is always the most appropriate tool to helping low- and moderate-income households post-disaster. For homeowners that really need a full indemnity policy, for example, perhaps a government means-tested assistance program with premium costs might be a better fit (e.g., National Research Council 2015; Dixon et al. 2017). Or, for households that would be able to take on debt, it may be more cost-effective to be given guaranteed access to credit. Currently, though, no means-tested assistance or credit guarantee programs exist in the U.S.

There is no precise definition of microinsurance. Sometimes it has been defined by the level of premium, other times by the level of coverage, and still others simply by being designed to reach poorer households. It is true, though, that in the United States, a microinsurance policy would probably need to offer a higher absolute payout than in developing countries, probably several thousand dollars of coverage at a minimum. While many of the disaster costs households experience are not property-damage, as discussed above, there is no comprehensive database of these costs. There is, however, information on property damage that can be instructive on the value of various coverage limits.

We obtained recipient level data on FEMA’s Individual and Households post-disaster grant program.^9^ This program, when authorized by a Presidential Disaster Declaration, makes small grants to qualifying households. As part of the intake process, the total damage of the applicant is assessed. In Table 2, we provide the median, 75^th^ percentile, and 95^th^ percentile of these damage estimates for a sampling of recent disaster events for households making less than $60,000 in income, broken down by renters and owners. The first thing to note is that damage can vary widely by disaster. Some disasters have highly skewed distributions and the severity of events clearly varies. Renters, as expected, experience lower absolute levels of damage. Looking across these, it is clear that a microinsurance policy can cover costs from smaller events, or those households with less severe damage, but clearly property-owners would need additional resources to fully fund repairs from severe disasters. A parametric microinsurance policy, then, should not be seen as a tool to fully cover all losses, but as a source of fast and flexible dollars to augment other assistance and boost recovery.

**Table 2:** Estimated damage to Individual and Household grant recipients earning less than $60,000 for select disasters, 2021 USD

Disaster	Median	75^th^ Percentile	95^th^ Percentile
Hurricane Harvey	Owner: $9,225 Renter: $2,154	Owner: $19,024 Renter: $4,614	Owner: $37,870 Renter: $8,643
Hurricane Sandy	Owner: $15,963 Renter: $3,467	Owner: $29,165 Renter: $6,342	Owner: $51,247 Renter: $9,794
Hurricane Ike	Owner: $2,995 Renter: $2,647	Owner: $10,709 Renter: $9,870	Owner: $48,028 Renter: $32,798
Louisiana flooding in 2016	Owner: $18,154 Renter: $3,953	Owner: $27,789 Renter: $6,850	Owner: $42,874 Renter: $10,838
California wildfires in 2018	Owner: $52,277 Renter: $7,167	Owner: $67,321 Renter: $9,769	Owner: $256,289 Renter: $14,372
Texas Ice Storm 2021	Owner: $3,597 Renter: $950	Owner: $4,018 Renter: $1,755	Owner: $6,770 Renter: $3,635

The premium of a policy will be directly linked, of course, with the likelihood and magnitude of the payout: greater payouts or higher likelihood of payouts will require larger premiums. What level of premium is affordable will depend on the specific population targeted. Those at the very bottom of the income distribution will likely need direct public assistance and may not be the best target group for microinsurance. Microinsurance may be more beneficially aimed at those that have limited means, but do have some source of moderately predictable income, for example. A detailed analysis of the target consumer group would be necessary for designing any product.

Finally, it is important to stress that there are also non-financial reasons a household might prefer a parametric insurance policy. For example, some moderate-income customers of a parametric earthquake product in the U.S. chose the option not just because it was more affordable, but also because they felt it shifted the power dynamic post-disaster: a parametric policy paid them quickly without time-consuming red tape or battles over the amount and left them free to best determine their highest needs for use of the funds.^10^ Post-disaster there has been reporting of challenges and delays households face in obtaining their insurance, for example, due to bureaucratic and regulatory hurdles that would not face a parametric product (for example: Newsday 2013). The speed of the payout and the freedom for the household to determine the best use of funds can make parametric microinsurance an important recovery boost to households that do not have savings or other sources for fast and flexible dollars.

## Challenges of Parametric Microinsurance in the United States

The third section documented the beneficial uses of parametric microinsurance and the likely roles it could play in the U.S. We turn now to the challenges this type of approach faces, discussing four hurdles identified in the literature review and interviews: (1) finding a profitable model or public sector support, (2) meeting regulatory requirements, (3) managing basis risk for the insured, and (4) overcoming a lack of demand.

### Finding a Profitable Model or Public Sector Support

Since the target population of microinsurance is lower-income households, it is absolutely necessary to keep premiums as low as possible in order to have a product they can afford to purchase—yet, this makes it much harder to find a potentially profitable and sustainable business model. As discussed above, a parametric product is likely essential, since it has lower transaction costs. While critical, further reductions in administrative costs will likely be needed to design a low-coverage and low-premium product that is viable (Biener and Eling 2012). Various models have been explored to lower costs—some discussed in more detail in the fifth section—from harnessing mobile technologies to partnering with other organizations in distribution. Microinsurance may benefit from community pricing, where a set premium is charged in an entire community, instead of underwriting different subgroups. This can help maintain affordability if the cross-subsidies do not create too much anti-selection; it is also much simpler to explain and administer and can help scale the product (Garand et al. 2012). Scaling can be critical for microinsurance to achieve a high enough volume, given low premiums, to reach profitability (Angove and Tande 2012). Various paths to scale have been explored in pilots around the world, such as by linking the microinsurance to other products or force-placing the cover through the use of a third-party organization (see “Distribution Channels and Delivery Models” section for more discussion).

It is also important to note that the start-up costs of the design process—including consumer research, hazard modeling, pricing, and developing policy terms—can be quite costly. In developing countries, lack of objective data for the trigger and limited historical data to use as an input to hazard modeling can present hurdles, but these will be less of a challenge in the United States. That said, globally the use of satellite and other remote sensing data has also made this less of a concern. Still, a needs assessment of the target population will need to be undertaken and detailed hazard and actuarial modeling completed, often in an iterative process of exploring triggers, coverages, and prices. It may be the case that the premium generated off microinsurance is too low to support a product design team; internationally, external teams funded by donors have sometimes played this role (Mapfumo et al. 2017).

This highlights that when targeting lower-income populations it may simply be the case that even after transaction costs have been reduced and attempts made to scale the product, to reach the targeted consumers, public or philanthropic support is necessary. Indeed, frequently around the world, microinsurance has been supported by the public sector or with philanthropic dollars, since profitability is a challenge, and microinsurance can unite insurance with clear social objectives (Hill et al. 2014). Public sector and philanthropic support can take many forms, including funding for product development, premium subsidies for target populations, or undertaking information provision and outreach (Surminski and Oramas-Dorta 2011; Warner et al. 2013; MCII 2021). Funding could also be accompanied by a more formal public–private partnership that encompasses many aspects of the process from design to delivery.

As microinsurance is adapted to the United States, then, an important question is whether it is a partially subsidized social safety net program, some new form of public–private partnership, or a standalone private sector business model. Since very few of the pilots of microinsurance that have been launched around the world have succeeded as standalone, long-term, private-sector business models, it seems more promising to explore how the public and private sectors can work together to provide financial resiliency for low-income households. Public sector funding for microinsurance programs need not necessarily require new funding vehicles. Existing disaster grant funding could potentially be harnessed for this purpose, such as programs through the Federal Emergency Management Agency (FEMA) or the Department of Housing and Urban Development. Congress, state legislatures, or local governments could also create an assistance program to provide premium-support to qualifying households to purchase microinsurance policies against disasters. Finally, it is conceivable that some of the foundations that have supported microinsurance in other countries would also be interested in supporting the launch of microinsurance for low-income populations in the United States, as well, such as by paying the up-front design costs.

### Meeting Regulatory Requirements

Unlike some other countries where microinsurance has been adopted, the United States has a highly developed and regulated insurance market. Parametric microinsurance will need to be designed to fit within this regulatory structure. In the U.S., insurance is regulated at a state level through offices of insurance commissioners, who focus on solvency and marketplace regulation, including consumer protections. Each state insurance commissioner would have jurisdiction over any microinsurance products in their state or territory. Some requirements, such as standard consumer protections, would likely apply to microinsurance, as they do to other insurance products. Other standards or regulations, such as solvency requirements, may need to be adjusted for firms that exclusively write microinsurance (Biener et al. 2014). But since both parametric and microinsurance are new to a U.S. residential market, commissioners will likely have some concerns and new guidance or approaches in certain areas may be needed. We undertook semi-structured interviews with several commissioners and their staff to better understand regulatory concerns in the U.S. market.

As noted in the introduction, Puerto Rico is the first U.S. state or territory to adopt regulations specifically for microinsurance. Many other countries, however, have also adopted regulation specifically for microinsurance as a class of products. In July 2020, Puerto Rico introduced new regulations for parametric microinsurance for catastrophes.^11^ Puerto Rico’s enabling of parametric micro-insurance products stems from its experience following Hurricane Maria in 2017 and the observed need for new insurance solutions tailored to the needs of low-income households. As of 2019, the U.S. Census estimated the Puerto Rican poverty rate at close to 43%. At the time of Hurricane Maria in 2017, less than 4% of housing units on Puerto Rico had flood insurance (Kousky and Lingle 2018).

The new regulations stipulate that the Insurance Commissioner’s Office must ensure that rates are not excessive, inadequate, unfairly unequal, or otherwise undermine the purpose of microinsurance. Therefore, rates are subject to a premium limit that should not exceed two percent of an individual’s annual income or the minimum wage ($7.25 per hour). For 2020, in order to consider a policy microinsurance, then, the premium could not exceed $261 per year or $21.75 per month. This premium limit is established for policies that cover only one catastrophic risk, but insurers can also offer policies with multi-peril coverage. Some other countries, such as Brazil, India, and Mexico have also defined microinsurance in terms of premium or coverage limits (Biener et al. 2014). Microinsurance premiums can be established for annual or monthly terms, allowing products to be offered for specific seasons. Insurance payouts must be made within 10 days of a triggering event. They policy document must also be simple, not exceeding four letter-sized pages in 12-point font. These regulations are so new, that at the time of writing, only one product was yet to market. The hope is that these regulations will provide the regulatory framework for market experimentation with microinsurance.

Two key issues for regulation emerged in our interviews—both of which were addressed in the Puerto Rican regulation. The first is that most state laws require insurance to indemnify an insured and thus require some type of “proof of loss.” Without this, the product would be considered a financial derivative^12^ and not insurance. Any approach to establishing proof-of-loss, however, must be rapid and inexpensive in order to not undermine the benefits of parametric designs. While not intended only for low-income households, one of the first residential parametric products on the market, Jumpstart,^13^ an earthquake policy in California, was able to meet this requirement with a simple text message from the insured that they have sustained costs as a result of the earthquake. Internationally, programs have made use of rapid assessment teams to quickly assess and verify damage, typically simply binning damage into one of only two or three categories (such as high or low); this could be used as a proof-of-loss and linked to either a high or low payout. Satellite images, drone data, or social media could also be used for this purpose (Kryvasheyeu et al. 2016). Alternatively, in Puerto Rico, they made the decision that any major hurricane that came through the island would inevitably cause some level of economic damage to residents and that the small payout from a microinsurance product could never be a financial windfall to the insured. As such, they completely waived all proof-of-loss requirements for microinsurance.

The second regulatory issue is one of distribution. In the developing world, as discussed more below, distribution is often done through channels that are not traditional approaches for selling insurance (Clyde & Co 2018). In the U.S., property insurance for households would typically be sold by licensed agents. In Puerto Rico, the new regulations created a new role of microinsurance distributor. This enables other individuals and entities beyond insurance agents to sell microinsurance policies. Other countries have also adopted different, often more relaxed, regulations for specific microinsurance agents or distributors (Biener et al. 2014). This allows for new approaches to distribution, as discussed in the fifth section.

There is a third topic that was not discussed in interviews, but for the U.S. context warrants discussion and that is whether microinsurance is an admitted product. In the U.S, there are two regulatory classes of insurance firms: admitted firms and surplus lines firms. Admitted carriers are licensed by the states in which they operate and file their rates and forms with the state regulator. In the case of insolvency, their claims are backed by state guaranty funds. Non-admitted carriers, also called surplus lines carriers or excess and surplus companies, though approved by the state, have no requirements on their rates and forms and are not backed by state guaranty funds, but they may have higher minimum solvency requirements than admitted carriers. Rate and form freedom allows them to specialize in nonstandard, unique, complex, or catastrophic risks. Since microinsurance is new to the U.S., it would likely need to first arise in the surplus lines market. However, low-income consumers are more vulnerable to an insurer insolvency, and thus may be more in need of the protection a state guarantee fund provides to consumers. It may be important, then, for any private market offering microinsurance to ultimately develop in the admitted market.

### Managing Basis Risk

As mentioned above, basis risk refers to the possibility that the payout may not be the same as total costs sustained by the insured. Since the payout is determined by a measure of the hazard itself and not the losses of the insured, the payout could be more or less than realized costs. Basis risk has been defined in the literature as a weak correlation between the trigger and individual losses (Clement et al. 2018). In practice, microinsurance is not designed to fully indemnify all losses, but, as discussed in “The Role for Parametric Microinsurance in the U.S.” section, to jumpstart recovery and assist with filling gaps in assistance. As such, total losses will often exceed the payout of a microinsurance policy. That said, a concern that we heard in the regulator interviews is the product failing to provide a payout when the consumer expected that it would; this could leave them more vulnerable if they failed to adopt other protective actions since they were assuming a payout for a particular type of event. This risk of damage with no payout also dampens demand (e.g., Elabed and Carter 2015; Jensen et al. 2018).

To prevent poor expectations, steps must be taken to guarantee consumers understand the concepts of parametric insurance and the details of the specific trigger design for the product they are purchasing (MCII 2021). Providers of parametric policies (microinsurance or otherwise), need to commit to designing their consumer-facing materials to help ensure that the trigger is transparent. We heard, for example, one useful way to do this is to use prior disaster events that are familiar to the consumer and demonstrate if they would have triggered the product or not. Clarity and transparency may be directly addressed by regulations. For instance, Puerto Rico’s microinsurance legislation requires that the policies:

"be drafted in precise, clear, and simple language, on no more than four (4) letter-size pages and clearly establishing the covered risks, exclusions, and other conditions that create rights and obligations, so that a lay person can understand the terms and conditions, without reference to clauses or covenants that are not contained in the policy. Specialized terms may not be used."^14^

Beyond simply managing expectations, basis risk should also be reduced to the extent practicable through careful attention to the design of the trigger. This can be done by choosing one or more measures that are known to be highly correlated with economic impacts and also by making tiered payouts. On the former, care must be taken to making sure that the data sources for the trigger are appropriately capturing conditions at the location of the insured, for example. On the latter, since it can be frustrating and difficult for consumers to “just miss” the cutoff for a payout, some products are designed to have payouts that increase depending on the intensity of the event. This way, the risk of having damage and no payout is reduced and consumers can obtain some demonstrated benefit of having the insurance even if the highest-level payout triggering conditions are not met.

### Overcoming a Lack of Demand

Regardless of the design, any microinsurance program will have to address the ongoing challenge of limited demand for disaster insurance, which exists at any income level. For some households, the lack of demand might be attributed to individual risk preferences and a rational choice given the price of insurance, competing uses of limited budget, and the ability to self-insure. For many other households, however, studies have shown low demand appears to be traced to other factors including lack of knowledge about disaster risks, insufficient financial and insurance literacy, lack of salience, well-documented behavioral biases when evaluating risks, mistrust of the firm or agency offering the product, concern the insurance does not meet individual needs, high transaction costs, peer effects, as well as budget and liquidity constraints (e.g., Patt et al. 2009; Clarke and Grenham 2013; Cole et al. 2013; Kunreuther et al. 2013; Platteau et al. 2017; Netusil et al. 2021). Many simultaneous approaches are likely necessary to overcome both price and non-price drivers of low demand. We highlight four of notable importance to a microinsurance market.

First, lower-income consumers may have less access to educational materials about risk and may be less familiar with insurance concepts. To overcome these hurdles, a well-developed consumer education campaign is likely needed and should be coupled to a very simple and easy-to-understand and easy-to-use product, as noted above. Given the dominance of indemnity-insurance in the U.S. market, regulators may request that product materials make clear that a parametric payout is made without regard to the damages that may be realized. To aid understanding about the product, consumers could be given information that will allow them to assess their losses under different disaster conditions and compare those losses to the parametric payout. Trust also drives demand (Patt et al. 2009; Cole et al. 2013); as such, outreach and product sales may be most influential when offered through trusted intermediaries already engaging with the target population.

Second, product design decisions that demonstrate value to consumers might spur demand. For instance, the value of the risk transfer function of insurance can be abstract; coupling insurance with some other product or service that produces a tangible benefit could help improve demand for the product. This could be a complementary product, or an annual rebate if no claim is filed. Prior work finds that demand for microinsurance often increases after observing payouts, likely related to both demonstrating value and trust in the institutions (Cole et al. 2014; Platteau et al. 2017). In addition, prior work in other countries has found that often people with low insurance literacy view insurance through a lens of balanced reciprocity and become disinclined to stay insured if many years pass with no payout, suggesting the need for products that pay more frequently, perhaps by covering multiple perils (Platteau et al. 2017). As such, insurance programs can be designed to provide some lower-level, but higher-frequency payouts, in addition to larger, but less frequent, payouts for catastrophic events. This can lead to more continual demonstration of the benefits of insurance, maintain insurance literacy, and test the claims management system for when a larger scale disaster does strike. That said, single peril coverage is much more common for microinsurance globally (Yore and Walker 2019) and products that pay more frequently will also be more expensive.

Third, studies from the developing world suggest that demand for parametric microinsurance falls as basis risk increases (Elabed and Carter 2015; Jensen et al. 2018). Many of the studies, though, are hypothetical surveys or inferred from choices where other determinants could have explained results (Clement et al. 2018). It is intuitive, however, that as the correlation between actual losses and payouts increases, the product should increase in value for consumers. As discussed in “Managing Basis Risk” section, this again highlights the importance of trigger design.

Finally, in many microinsurance schemes around the world, the challenges with low demand are overcome by some amount of force-placed coverage. For example, insurance may come packaged with another valuable product—often credit. Participation may also be incentivized through subsidization of the premiums. While premium subsidies may be justified as assistance for lower-income households, it also raises questions about long-term sustainability if the support ends.

## Distribution Channels and Delivery Models

Distribution of insurance refers to all the activities that must take place between the holder of the risk and the client, including policy origination, collecting premiums, marketing, sales, and claims payments (Smith et al. 2012). This could involve multiple partners beyond just the insurance company. One key lesson from international efforts is that it “takes time (sometimes years) for the ultimate beneficiaries of index insurance products to begin to truly appreciate the benefits of the cover, for delivery channels to build their sales and administration capacity and for (re)insurers to adjust and improve the product so as to better attend to client demand,” (Bernhardt 2014). This is likely to apply in the U.S., as well: full development of workable microinsurance models could take many years.

In this section, we provide a conceptual overview of four delivery models that have the potential to be used in the U.S. market to secure the benefits of microinsurance. All of them, as we discuss, help solve one or more of the challenges of offering parametric microinsurance discussed in the fourth section. Choosing which model to pursue would require a detailed investigation of which model best fits a specific peril and insured population. As noted above, before designing and pricing a microinsurance product, it is first necessary to understand the recovery needs of the target population for the particular hazard, including the type and range of costs they might incur post-disaster, other sources of financial support, and their previous coping strategies (Garand et al. 2012). The investigation needs to include the legal and regulatory barriers and opportunities and the potential governmental, NGO, and private sector interest in delivering the product. In practice, different models could operate simultaneously for different target groups and different perils.

### Aggregator Model

**Fig. 3:**
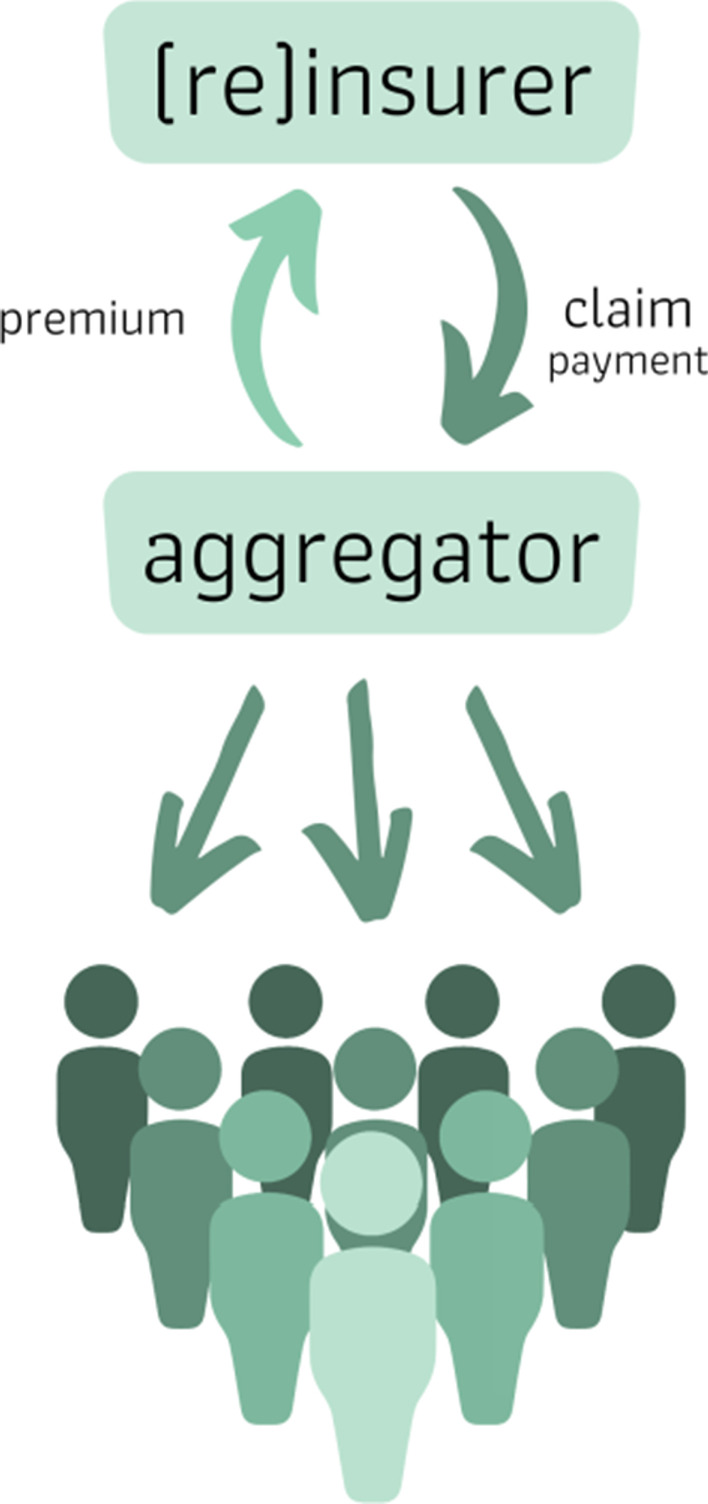
Aggregator model

The first model we discuss is one in which another institution, referred to as the aggregator, purchases a single, larger policy, but then then disburses the claim payment to the individual households. The aggregator may be a community non-profit, a local governmental agency, or a disaster relief NGO. This model is sometimes referred to as a “meso-level” model. In this approach, the aggregator is the intermediary between the insureds and the (re)insurance firm providing the coverage. The aggregator negotiates an insurance contract with the (re)insurer and holds the policy. The aggregator secures the funds needed to pay the premium from the insured and perhaps from other sources. Figure 3 provides a schematic of this model.

Potential aggregators are often those whose social goals can also be achieved through insurance for the populations they serve. This could be an NGO focused on poverty reduction, disaster recovery, or housing affordability, for example. Such an NGO likely already has trust in the community, which is valuable when introducing a new concept and product, as microinsurance would be to most of the U.S. A public sector agency could also play the role of an aggregator. A public agency could support the policy in other ways, as well, such as through premium support or with accompanying risk reduction and/or education programs.

There is substantial flexibility in how claim payments could be made. Disbursement could be done by simply paying a set amount to all those impacted, or it could be done through visits to assess damage, or by examining satellite or aerial photographs to determine damage, for example. Amounts paid could also vary by income of the impacted household, or other metrics. To limit basis risk, the aggregator would need to have a process for allocating more funds to those with higher damage levels. Note, that to be considered microinsurance, however, the households should be aware of their coverage and the disbursement process before the disaster occurs. Presumably, they would be paying at least some portion of the premium to the aggregator, even if partially covered by government or philanthropic funds. If that if the households paid no premiums, and the disbursement process was not determined ex-ante, but was simply determined by the aggregator post-disaster, then this model would morph from being microinsurance to instead being an aid program administered by the aggregator and financed through insurance. While potentially beneficial, this would not strictly be microinsurance, since the households would not have any pre-disaster guarantee or knowledge of the coverage.

The aggregator model solves the challenges discussed in the fourth section. First, it helps reduce costs, which improves affordability and helps create a sustainable business model. It is also an effective structure for a public–private partnership. This model can also minimize basis risk by having the aggregator examine post-disaster losses and distribute funds to those most in need. The model overcomes the issue of lack of demand since the aggregator secures coverage on behalf of a larger group. The model also introduces no additional regulatory hurdles beyond those for parametric policies more broadly.

### Mobile Model

The expansion of mobile phone use has created the possibility of mobile-based business models for microinsurance. As of 2019, around 96% of Americans owned a phone, with 81% of people having a smartphone; among adults making less than $30,000, 95% still had a cell phone and 71% had a smartphone (Pew Research 2019). In mobile-based models, an insurer offers policies directly to households through a mobile application. Mobile technologies can allow for policies to be purchased, premiums paid, claims received, and can also be used for consumer communication and education. Smartphone photos or text messages could be used to meet proof-of-loss requirements. Mobile-based technologies can address some of the challenges in the fourth section. They can reduce transaction and administrative costs and grow the risk pool by expanding the geographic scope over which policies are offered. This can help make a more profitable model and expand demand. Mobile-based approaches are just one example of how technology is transforming the insurance sector, including microinsurance (e.g., Smit et al. 2017). These approaches, if involving new distributors, may need additional regulatory action, as discussed above it. Mobile models may not provide any additional benefits in terms of reducing basis risk.

Using mobile phones to distribute insurance builds on the development of mobile-based financial transactions. Smart card technology or mobile money platforms (such as M-PESA,^15^ originally launched in Kenya) have allowed for premium and claims payments among even the unbanked in other countries around the world. These types of platforms allow the user to store, send, and receive funds on a mobile phone. While the percentages of households that are unbanked in the U.S. is much smaller than elsewhere—estimated at just over 5% in 2019 (FDIC 2020)—mobile wallet platforms could also be harnessed in the United States to offer microinsurance to this population. And for those that are banked, platforms like Venmo, which have increased in usage in the United States, could theoretically be harnessed for microinsurance, as well. Mobile phone applications lower operational costs and reduce inefficiencies for insurers, allowing them to potentially offer many low-premium policies at a high volume, making microinsurance more financially viable.

Mobile-based technologies have already been used in the insurance sector in the U.S. for standard property insurance. One example, for instance, is the company Lemonade. Lemonade delivers insurance policies and handles claims through desktop and mobile apps using chatbots. There has been a rapid increase globally in the number of mobile microinsurance products across product areas like life, health, accident, cattle, crop, and travel insurance (Tellex-Merchan and Zetterli 2014). Many of these in the developing world are done in partnership with mobile network operators. In the U.S., a mobile-based microinsurance product could be done via an app on smartphones and not necessarily involve a mobile carrier.

Mobile-based models can have other benefits. Following Hurricanes Maria in Puerto Rico or Harvey in Texas, for example, many of those affected lost essential documents such as insurance policy papers, land ownership records, and personal identification needed to file a claim; these could be stored in an app for easier claim processing. These applications could also provide more value-add services and benefits that can improve the risk management practices of clients, like localized weather forecasts and updated information about claims processes.

### Joint Product Model and Joint Sale Model

The third distribution model is for an insurer to partner with another firm either to automatically couple sale of the insurance to another product, or to simply make the insurance product available at the time the consumer is buying something else. These types of partnerships can vary in structure. The involvement of the partner could range from passively making the insurance available at the point another product is bought to providing information on the product or attaching it to sale of their product. The benefit of these partnerships is that the insurer has access to a much wider customer base. A drawback can be that the partners may not know much about insurance or be effective educators about the product to consumers.

In the first version, a commercial enterprise finds it attractive to increase insurance penetration among its customers, or finds is worthwhile to allow insurance to be bundled to their product as part of a social service. For instance, perhaps landlords require a microinsurance product as part of signing a lease, knowing this will then lessen the likelihood a tenant misses a payment post-disaster. It could also be the case that a public sector program finds coupling the insurance to the program beneficial for recipients, perhaps partially supporting the purchase with public funds. For example, the Low Income Home Energy Assistance Program could also purchase a parametric microinsurance policy for their beneficiaries. This approach solves the demand challenge by essentially forcing coverage on certain groups. This can also help expand the risk pool to create a more profitable model.

In the second version of this model, an insurer partners with one or more retail outlets or other firms to sell the microinsurance policy. The key benefit of this approach is access to a larger potential client base, although purchase of the product is voluntary. It can also lower the costs of outreach and distribution, by drawing on an already existing network. Experience from other countries suggests that insurers may want to partner with many firms to sell their product in order to develop broad access to customers and successfully expand demand (Smith et al. 2012).

Partner firms may be compensated for selling the coverage. In order to keep fees low, however, it may be preferable if the partner also benefits in some way from the insurance, such that they are willing to sell the coverage, even if not highly compensated for doing so. For example, the insurance coverage could include paying an insured’s utility bill in the event of a disaster, such that a utility company would also benefit from helping market the product, or could include a certain amount to pay off credit card debt and thus would also be offered with taking out a credit card. It also works better for insurance purchase if the partner is a well-trusted brand (Smith et al. 2012). This helps keep the product affordable and demand high.

### Public Sector Insurance Program

In the United States there exist many quasi- to fully public disaster insurance programs. At the federal level this includes the National Flood Insurance Program (NFIP), which writes standalone flood policies to members of participating communities (Kousky 2018). All states in the southeast U.S. exposed to hurricane risk have residual market mechanisms, also called wind pools or beach plans, which offer wind coverage to those unable to secure coverage in the voluntary market (Kousky 2011; Hornstein 2016). The state of California has the California Earthquake Authority (Marshall 2018) and also uses its Fair Access to Insurance Requirements (FAIR) plan to cover wildfire risk. Such government disaster insurance programs exist in almost all developed countries (e.g., McAneney et al. 2016).

Any of these public sector disaster insurance programs could also offer a parametric microinsurance policy. For example, the existing state programs or the NFIP, in addition to the standard policies they offer, could make available a microinsurance policy for residents of participating communities that meet pre-determined income criteria. The payouts could be offered as flat payments in the case of loss, such as $5,000 or $10,000, or could be designed as two or three tiers depending on the severity of the event. For the NFIP, Congress could work with FEMA to make microinsurance policies that would satisfy the mandatory purchase requirement for those who meet a certain income level, such as those below a certain percentage of area median income or those below the federal poverty level. Alternatively, a state, a local community or association of local communities might stand up a new program specifically to offer parametric microinsurance.

Using a public sector program can overcome some of the challenges identified. First, affordability could be supported by public dollars. Any microinsurance policy could also be coordinated with other social support programs. If coupled with other activities, such a post-disaster damage assessments, it may be possible to reduce basis risk without substantially increasing policy costs by having more tailored payouts. Lack of demand may, though, still be a challenge unless some form of mandate was adopted.

Another potential public sector approach is a municipal captive. A captive insurance company is one that is wholly owned and controlled by the insureds and used to provide coverage for their own risks. Like any other insurance company in the U.S., captives are regulated by state departments of insurance. Captives tend to be created when insurance is difficult to obtain or is too expensive in the private market. They retain premium and directly access reinsurance markets. There are many different structures for captives (WillisTowersWatson 2011). Several public-sector entities have captives, from school districts and utilities to a few municipalities. Theoretically, a municipal captive could be used to write microinsurance policies directly to lower-income households in the municipality’s jurisdiction, although this likely requires enabling legislation. Any accumulated revenue could be used to invest in risk reduction measures targeted at the properties and neighborhoods being offered the coverage. Given that the captive is controlled by the local government, they could also couple the insurance to social programs or subsidize the policies with public funds. Such funds may be needed to initially capitalize the captive for offering the policies, for example. While a captive provides an existing insurance structure that might be harnessed for microinsurance, establishing a captive initially is not an easy or inexpensive undertaking. It may not prove cost-effective if done only for the microinsurance line. A detailed feasibility study would need to be undertaken for any municipality considering formation of a captive.

## Conclusion and Next Steps

This paper reviewed the evidence and provided a proof-of-concept examination of utilizing parametric microinsurance in the United States to improve the financial resiliency of lower-income households, with lessons for other developed nations. Lower-income households are disproportionally harmed by disasters and struggle financially post-disaster to fund the necessary repairs and with meeting other unexpected disaster costs. This can have negative spillover impacts into many other areas of well-being. Parametric microinsurance has proved a viable approach to improving the financial resilience of lower-income households in other locations around the globe. There are three key benefits associated with microinsurance: it can be affordable enough for lower-income populations, payouts are typically very rapid, and the dollars can be used flexibly for any post-disaster need. This review also identified four challenges with making microinsurance available: (1) finding a profitable model, while keeping premiums low, or finding public or philanthropic support for the microinsurance, (2) managing the basis risk for vulnerable populations, (3) overcoming lack of demand, and (4) meeting regulatory requirements. These are summarized in Table 3. We identified four promising delivery models that could be adapted for a U.S. context and which would overcome at least some of the identified challenges. The models discussed include providing microinsurance (1) through an aggregator, (2) through a mobile-based application, (3) as a joint product or joint sale, or (4) through a public-sector disaster insurance program.

**Table 3:** Benefits and challenges for parametric microinsurance in the United States

Benefits	Challenges
Affordable	Identifying a profitable model while keeping premiums low or finding public sector/philanthropic support
Fast payment	Managing basis risk
Flexible use of funds	Lack of demand
	Meeting regulatory requirements

While these four models hold potential, further research and development will be needed, along with pilots to test hypotheses and implementation details. Several priority areas for research and development that could speed implementation are suggested by this proof-of-concept review. First, while there is a robust literature on the differential impacts of disasters on disadvantaged communities, none of this research documents in detail the specific financial costs faced by various households for different perils, their current sources of support, and the gap in post-disaster financing that needs to be closed. Such needs assessment research will be necessary for development of useful and robust microinsurance programs.

Second, the scope and form of regulation that protects consumers, while facilitating the offer of this product and stimulating supply, needs further development. Puerto Rico is leading the effort on this and other jurisdictions will need to explore the possibility, as well. Regulators also oversee insurance agents and will need to identify any regulatory requirements if insurance distribution for microinsurance policies bypasses these agents or is undertaken by others, as suggested in some of the models in the fifth section. Another concern of regulators is consumer understanding. More research is needed on the financial and insurance literacy of potential customers and on the best approaches for education about the role of parametric insurance, its triggers, and payout structure.

Parametric microinsurance is a tool to improve the resilience of some of the most vulnerable households. As such, it would benefit from public support. So, finally, policymakers, working with researchers and all stakeholders, will need to explore what role this should take going forward. For instance, is there public funding to help cover the cost of premiums for certain groups? Or funds to cover the development and piloting phase of a microinsurance program? Could the public sector partner in outreach and education? Beyond the public sector, are there philanthropic donors that could help develop this concept? For example, the Bill and Melinda Gates Foundation provides tens of millions of dollars in funding to launch the ILO’s Microinsurance Innovation Facility and expand microinsurance in many developing countries.^16^ These conversations need to begin in a U.S. context.

As with all forms of risk transfer, insurance is most powerful in building resilience when tightly linked to both risk reduction, risk communication, and disaster preparedness and recovery. We need robust integrated risk management strategies to ensure that those located in high risk areas, particularly those that are low income, are better equipped to be financially resilient after disasters. Officials need to work together across all levels of government and with non-governmental organizations and the private sector to create new and innovative solutions to help fill gaps in disaster preparedness and recovery.

## Footnotes

1 See: https://riskcenter.wharton.upenn.edu/digital-dialogues/improvingdisasterrecovery/

2 Beyond recovery, microinsurance can play an important role in allowing risk-taking activities that would increase income or assets, and thus help lift people out of poverty, such as when crop insurance allows for the planting of riskier, but higher-revenue crops.

3 See https://data.census.gov/cedsci/table?q=homeownership&tid=ACSST1Y2019.S2502.

4 See: https://www.swissre.com/institute/research/sigma-research/sigma-2020-04/us-canada.html

5 See: https://www.ncdc.noaa.gov/billions/

6 Calculated using publicly available data on disaster declarations from OpenFEMA: Disaster Declarations Summaries – v2, U.S. Federal Emergency Management Agency. Accessed October 28, 2020. https://www.fema.gov/openfema-data-page/disaster-declarations-summaries-v2.

7 Extremely low-income is defined as 30% or less of area median income, very low-income is defined as 31% to 50% of area median income, and low-income is defined as 51% to 80% of area median income.

8 The median income of manufactured home residents is roughly half the median income of those living in standard site-built housing, with a quarter of these residents earning less than $20,000 annually (Zahalak 2020).

9 OpenFEMA Dataset: Individuals and Households Program—Valid Registrations—v1, U.S. Federal Emergency Management Agency. Accessed March 16, 2021. https://www.fema.gov/openfema-data-page/individuals-and-households-program-valid-registrations-v1

10 Personal communication with Kate Stilwell, CEO of Jumpstart.

11 Rule No. 103 of the Regulations of the Insurance Code of Puerto Rico "Requirements for Submitting and Processing Parametric Catastrophe Microinsurance in Personal Lines.".

12 Derivatives are financial securities the value of which hinge on the value of an underlying asset or index. In the U.S., derivatives are regulated by the Commodity Futures Trading Commission, the Securities and Exchange Commission, and Financial Industry Regulatory Authority and have different tax treatment than insurance.

13 See: https://www.jumpstartrecovery.com/

14 *Rule No. 103 of the Regulations of the Insurance Code of Puerto Rico "Requirements for Submitting and Processing Parametric Catastrophic Microinsurance in Personal Lines."* Office of the Commissioner of Insurance of Puerto Rico, Government of Puerto Rico. July 2, 2020.

15 See more details at: https://www.worldremit.com/en/kenya/mobile-money/m-pesa

16 See: https://www.ilo.org/global/about-the-ilo/newsroom/news/WCMS_088398/lang--en/index.htm.
